# Neurological dysfunction screening in a cohort of adolescents with type 1 diabetes: a six-year follow-up

**DOI:** 10.3389/fmed.2024.1331145

**Published:** 2024-05-09

**Authors:** Davide Tinti, Carlotta Canavese, Cecilia Nobili, Daniele Marcotulli, Erika Daniele, Ivana Rabbone, Luisa de Sanctis

**Affiliations:** ^1^Department of Pediatrics, A.O.U. Città della Salute e della Scienza di Torino, Turin, Italy; ^2^Child and Adolescent Neuropsychiatry Unit, Department of Sciences of Public Health and Pediatrics, University of Turin, Turin, Italy; ^3^Postgraduate School of Pediatrics, University of Torino, Torino, Italy; ^4^Department of Health and Science, University of Piemonte Orientale, Novara, Italy

**Keywords:** diabetes, adolescent, hypoglycemia, nerve conduction study, diabetic neuropathy

## Abstract

**Aims:**

Diabetic neuropathy (DN) is one of the most insidious microvascular complications in patients with type 1 diabetes (T1DM) and initial signs may appear during childhood. The aim of this study is to evaluate associations between the Nerve Conduction Studies (NCS) outcomes at enrollment with neuropathy screening questionnaires performed six years later in a cohort of asymptomatic adolescents followed up until early adulthood, affected by T1DM.

**Methods:**

We performed NCS in a cohort of seventy-two adolescents with T1DM and eighteen healthy controls. Six years later, screening questionnaires for DN were proposed: Michigan Neuropathy Screening Instrument (MNSI, specific for symptoms of somatic dysfunction), Composite Autonomic Symptom Score 31 (COMPASS 31, specific for abnormalities of the autonomic component) and Clarke questionnaire (perception of hypoglycemia). Thirty-two TD1M subjects agreed to participate in the follow-up; main clinical-metabolic parameters, including the number of episodes of hypoglycemia in the past twelve months, were collected.

**Results:**

11.8% of subjects showed changes compatible with DN through the MNSI questionnaire, while 41% declared a reduced perception of hypoglycemia on the Clarke questionnaire. No significant correlation was observed between the clinical-metabolic parameters or altered response to NCS and scores of MNSI and COMPASS 31 questionnaires. On the other hand, an association was observed between NCS abnormalities and a high number of hypoglycemic events after six years (97-fold increased risk, *p* = 0.009).

**Conclusion:**

The frequency of somatic alterations in the study population is 11.8%, whereas the frequency of symptoms correlated with autonomic damage is about 41%. An autonomic impairment recorded at NCS may represent a six-year risk factor for increased hypoglycemic episodes, even if more extensive studies are needed to investigate this possible relationship further.

## Introduction

Type I diabetes mellitus (T1DM) is a complex metabolic disease characterized by autoimmune-based destruction of pancreatic beta cells and subsequent insulin deficiency (1).

Chronic complications of the disease result in reduced quality of life for patients and represent one of the most significant healthcare costs (2). Therefore, prevention in this field plays a pivotal role in the treatment and follow-up of these patients (3).

Diabetic neuropathy (DN) is part of the chronic microangiopathic complications and is associated with high morbidity and mortality. During the pediatric age, the clinical presentation of this complication is minor, but it is possible that some symptoms may begin during puberty, especially for long illness duration (4). The epidemiology of DN in the pediatric population needs to be better defined in the literature due to the need for studies of sufficiently large samples of children and adolescents. The prevalence is highly variable in different studies, depending on the specific age groups considered: the US SEARCH study reported a DPN prevalence in T1DM adolescents of 8.5%, and a worldwide meta-analysis reported a prevalence of 17.5% for all patients with T1DM (5, 6).

The etiopathogenesis is mainly related to prolonged exposure to high blood glucose levels since hyperglycemia increases intracellular production of sorbitol with subsequent myo-inositol reduction. In addition, glucose availability facilitates protein glycosylation, leading to an endothelial injury, especially in smaller vessels, altering the blood supply to nerve endings. Myelin may also be affected by these glycosylation phenomena, exposing it to macrophage phagocytosis with segmental myelin loss (7). These phenomena are then compounded by several other causes found in the literature, including advanced glycosylation end products (AGEs) leading to involutional phenomena with axonal loss, small vessel damage due to persistent hypoxic conditions, genetic predisposition, and the presence of autoantibodies directed against vagal and sympathetic ganglion components (8, 9).

DN is a pathological condition that can affect both the somatic and autonomic nervous systems, leading to a very heterogeneous spectrum of manifestations. The most common form is represented by sensorimotor polyneuropathy, while the focal form is extremely rare in pediatric age. Typically, the damage proceeds by first altering the smaller-calibre fibers and continuing only later to the large motor fibers; for this reason, the initial symptomatology is usually sensory (10). In contrast, the autonomic form is more frequent and may occur in patients with relatively short disease duration. The most frequent symptoms of autonomic neuropathy involve the cardiovascular, gastrointestinal, and genitourinary systems, often associated with metabolic, sudomotor and pupillary manifestations (11).

DN is still a clinical diagnosis, as the onset of symptoms allows the detection of this complication. Its screening usually starts at age 11 in individuals with at least 2–5 years of disease and is based on history, physical examination, and clinical tests performed annually (3). However, the neural damage evolves over many years, during which the patient is subjectively asymptomatic; tracing the complication at this stage would significantly improve the chances of intervention.

One tool for this purpose is the nerve conduction study (NCS), which represents the gold standard for the diagnosis of DN. It is a minimally invasive examination using surface electrodes and is, therefore, also suitable for pediatric evaluation.

The primary objective of the present study was to assess the presence of sensory, motor, and autonomic nerve conduction abnormalities in a cohort of children with T1DM through electrophysiological studies and subsequently correlate these data with the development of clinical symptomatology attributable to DN over the years. A secondary objective of the study is to correlate neuropathy with patients’ T1DM metrics.

## Subjects and methods

### Patients

Adolescents (12–18 years) with T1DM followed at the Centre of Pediatric Diabetology in the A.O.U. Città della Salute e della Scienza Children’s Hospital (Regina Margherita) in Turin, Italy, were invited to participate in this study from September 1, 2014, to March 16, 2015.

We invited patients coming from a group that appears to be at higher risk of microangiopathic complications in the pediatric age (12), according to the following inclusion criteria:

- diagnosis of T1DM according to ISPAD 2018 criteria (1)
- age between 12 and 18 years at the time of recruitment
- adult-type pubertal stage (P5, according to Tanner’s classification)
- duration of diabetic disease ≥5 years
- absence of clinical signs and/or symptoms of DN
- intensive basal-bolus type insulin treatment uninterrupted since the onset of T1DM.

Instead, the exclusion criteria were as follows:

- pubertal stage (according to Tanner) less than 5
- duration of T1DM <5 years at recruitment
- presence of suspected or confirmed clinical signs and/or symptoms of DN
- associated disorders and/or treatments with potential action on the peripheral nervous system (e.g., anticholinergic drugs).

Anamnestic data, including neurological diseases and cardiovascular risk factors (such as dyslipidemia, hypertension, or genetic factors), were collected for each participant. In addition, objective examination with weight, height, and BMI (with the calculation of respective centiles according to Cacciari et al.) was performed (13).

Age of onset, duration of disease, presence of ketoacidosis at onset, C-peptide assay, HLA haplotype, IA2 and GAD antibody status, type of insulin therapy (pens or insulin pumps), carbohydrate count (if any), daily insulin requirement, adherence to proper dietary norms, and performance of adequate physical activity were also assessed.

Glyco-metabolic compensation was then assessed by evaluating glycated haemoglobin at the time of recruitment and over the past two years using HPLC (high-pressure liquid chromatography). Finally, the simultaneous presence of other microangiopathic complications was checked by evaluating fundus oculi photography for diabetic retinopathy screening, and the albumin/creatinine ratio in 3 subsequent urinary spots of the first-morning urine was made to rule out diabetic nephropathy.

Healthy children have been included in the study to have controls according to the age and sex of our study population.

### Electrophysiological studies

Nerve conduction studies (NCSs) were performed by trained electromyographers with a Viking^™^ electromyography apparatus (Nicolet^®^ EDX, Natus Medical Inc., CA). Standard neurophysiological techniques using surface electrodes were adopted for all recordings, with standard values derived from the literature (14). We evaluated the median motor and sensory nerve, ulnar sensory nerve, motor external popliteal sciatic nerve, motor internal popliteal sciatic nerve, and sensory sural nerve. The variables recorded were motor nerve conduction velocities, compound muscle action potentials, sensory nerve conduction velocities and sensory action potential amplitudes. F-wave latency and conduction velocity were evaluated for the tibial nerve. Sympathetic skin response (SSR) was used to evaluate autonomic nerve function. Standard silver-silver chloride surface electrodes were used. The active recording electrodes were attached to the palmar surfaces of the hands and on the plantar surfaces of the feet, with the reference electrodes on the dorsal surfaces of the hands and feet, respectively. The stimulating electrodes were placed over the median nerve at the wrist and the tibial nerve at the ankle; the ground electrode was located on the stimulated limb. The SSRs for the four limbs were derived simultaneously. As the SSR habituated with subsequent stimulation, the stimuli were given at an irregular frequency of <0.1 Hz. All recordings were conducted using the described electrode position. Supramaximal stimuli were used to obtain meaningful responses. The response was considered absent if no voltage change was observed after two or more trials.

### Statistical analysis

Continuous variables are presented as means and standard deviation if normally distributed and compared using the Student t-test, as medians and interquartile ranges if non-normally distributed and compared with the Mann–Whitney U test. Categorical variables were compared with the chi-square test. Correlation analysis was performed using linear regression and Poisson regression. Analyses were conducted using R software (R Foundation for Statistical Computing).

### Follow-up questionnaires

All recruited subjects were contacted after a 6-year interval to undergo a questionnaire designed to identify the presence of clinical symptoms attributable to DN. The questionnaire included a brief clinical-anamnestic evaluation and the validated MNSI, COMPASS 31, and Clarke questionnaires.

To inform patients and their parents/guardians about the continuation of the present study and collect their adherence, they were contacted by telephone; subsequently, those who had expressed their willingness to participate signed the informed consent form. Finally, questionnaires were sent out for self-completion after appropriate online platforms were constructed.

The first part requested general information to compare the anamnestic data found in the initial phase of the study with the patients’ current condition. Specifically, the following information were requested: the values of the last three Hb1Ac and their respective dates; insulin-therapy regimen together with daily insulin requirement; the occurrence of a diagnosis of diabetic nephropathy, diabetic retinopathy, cardiovascular complications, or other T1DM-related complications; the occurrence of other autoimmune diseases; the number of episodes of severe hypoglycemia or ketoacidosis events in the last year; the performance of additional electroneurographic investigations and their report; hours of physical activity per week (defined as inadequate if <1 h/week, fair if between 1 and 5 h/week, and adequate if >5 h/week); and adherence to the dietary pattern (defined as inadequate, fair, or adequate). After this brief history, the validated MNSI, COMPASS 31 and Clarke questionnaires were administered.

The MNSI questionnaire was aimed to identify clinical symptoms that can be correlated with the presence of sensorimotor peripheral polyneuropathy. It was scored according to the indications reported in the literature (15). For each “Yes” answer to questions 1–3, 5–6, 8–9, 11–12, and 14–15, 1 point was attributed; for the “No” answer to questions 7 and 13, 1 point was attributed. Question 4 allowed assessment of circulation efficiency and question 10 gave a general indication of the level of asthenia; neither was included in the score. The sum of the individual scores obtained gives the final score. A total score ≥ 3 was considered pathological. To evaluate the frequency of sensory and motor conduction impairment in these subjects, the percentage of subjects with a positive result on the questionnaire was then identified.

The COMPASS 31 questionnaire was used to identify symptoms correlated with autonomic damage in individuals with diabetes mellitus. The score was calculated by assigning a value to each response according to the reference table. The final score was then calculated by multiplying the total result of each domain by a specific coefficient that assesses the weight of each portion of the questionnaire on the probability of being affected by DN (16).

Finally, the questionnaire defined by Clarke et al. investigates subjects’ awareness during hypoglycemic episodes; its score identifies three categories: (A) full awareness, (R) reduced awareness and (U) total unawareness. The final score identifies a condition of total unawareness if the defined response (U) is present, reduced awareness if there are at least four responses (R); if these criteria are not met, the subject is considered to be fully aware (A) of his hypoglycemic episodes; if these criteria are not met, the subject is considered to be fully aware (A) of his hypoglycemic episodes (17). The modified Clarke questionnaire gives another possibility in scoring; in this case, the score is given on a numerical scale. A higher probability of altered perception of hypoglycemic states is considered for scores ≥10; this scale has allowed for more accurate tracking of subjects with an initial reduction in perception (18).

After obtaining the information just described, we assessed whether a correlation existed between the variables under study. The association between questionnaire results and outcome of nerve conduction study examination, diet adequacy, and BMI was then evaluated. Since Clarke’s questionnaire investigates the level of awareness during hypoglycemic events, the correlation between the number of severe hypoglycemias in the past 12 months and the parameters just mentioned was also analyzed.

## Results

72 adolescents with T1DM (36 male and 36 female, mean age 15.91 ± 1.62 years) and 18 age-sex-matched healthy controls (12 female and 6 male) were enrolled in the study.

Data collected from the NCS examination showed that all study subjects had sensory-motor conduction parameters in the normal range. At the same time, evaluation of cutaneous sympathetic response (SSR) demonstrated that 26.38% (19 out of 72) of T1DM patients manifested an absence of lower limb signal. On the contrary, NCS data collected from healthy adolescents were in the normal range for both the sensorimotor component and SSR, as shown in Table 1.

**Table 1:** Average and standard deviation of the main parameters of motor and sensory components for each nerve studied in 72 adolescents with T1DM and normal SSR (group 1) and in 18 healthy adolescents (controls).

Sensory-motor component		Patients (*N* = 72)		Controls (*N* = 18)		
		Average	SD	Average	SD	*p*-values
Median motor nerve	Amplitude (mV)	10.46	2.91	10.77	5.42	ns
	Velocity (m/s)	55.64	4.22	58.39	3.4	0.02
Median sensory nerve	Amplitude (μV)	49.74	14.34	54.915	16.89	ns
	Velocity (m/s)	58.68	5.44	63.25	6.22	0.02
Sensory ulnar nerve	Amplitude (μV)	42.58	14.38	45.39	14	ns
	Velocity (m/s)	59.63	4.34	63.77	5.7	ns
Motor external popliteal sciatic nerve	Amplitude (mV)	5.89	1.84	5.11	1.06	0.04
	Velocity (m/s)	48.46	3.01	50.69	2.415	0.01
Motor internal popliteal sciatic nerve	Amplitude (mV)	11.89	3.45	12.6	2.57	ns
	Velocity (m/s)	45.85	2.61	50.61	3.2	<0.001
Sensory sural nerve	Amplitude (μV)	21.87	10.11	22.28	8.0	ns
	Velocity (m/s)	52.83	6.28	56.5	4.81	0.02

*p*-value is calculated using Student’s t-test.

As shown in Table 1, the collected data analyzed by Student’s t-test demonstrate statistically significant differences between the two groups. Specifically, while remaining in the normal range, a reduction in motor conduction velocity (MCV) of the sciatic external popliteal and sciatic internal popliteal nerves, sensory conduction velocity (SCV) of the median, ulnar, and sural nerves, F-wave latency, and sensory components of the median and ulnar nerves were observed.

This finding allowed us to divide the sample of our patients with T1DM into two groups characterized by the presence (Group 1, 53 patients) or absence (Group 2, 19 patients) of SSR response. A case–control comparison was made between the two groups. Clinical data are shown in Tables 2, 3.

**Table 2:** Median and interquartile range of the main clinical parameters for patients resulted with normal and altered SSR response.

		Group 1 (*N* = 53)		Group 2 (*N* = 19)		
		Median	IQR	Median	IQR	*p*-values
General	Onset (years)	8.1	6–9.8	6.7	4.5–8.8	ns
	Diabetes duration (years)	8.0	6.2–10.3	8.3	57.6–10.4	ns
	Weight (kg)	61	56–67	60	53–69	ns
	Height (m)	1.6	1.6–1.7	1.7	1.6–1.7	ns
	BMI	22	20–24	21	21–25	ns
Metabolic outcome	HbA1c (%)	7.9	7.3–8.6	7.5	7.1–7.8	ns
	Mean HbA1c last year (%)	7.8	7.3–8.5	7.6	7.3–8.0	ns
	Mean HbA1c last 2 years (%)	7.9	7.4–8.5	7.5	7.2–8.0	ns
Insulin therapy	Total daily dose (UI)	58	45–68	54	46–72	ns
	UI/kg	0.95	0.81–1.06	0.92	0.86–0.99	ns

*p*-value was calculated using Mann–Whitney U test.

**Table 3:** Median and interquartile range (IQR) of the main clinical parameters for patients resulted with normal and altered nerve study.

		Group 1 (*N* = 53)		Group 2 (*N* = 19)		
		%	*n*	%	*n*	*p*-values
General	Sex (M)	49	26	53%	10	ns
Comorbidities	Thyroiditis	19	10	11	2	ns
	Celiac	2	1	0	0	ns
	Early-stage retinopathy	11	6	11	2	ns
	Microalbuminuria	13	7	5	1	ns
Onset	Diabetic ketoacidosis	23	12	26	5	ns
	IA2 antibody	74	25	85	11	ns
	GAD antibody	62	21	69	9	ns
Insulin therapy	Carbohydrates counters	60	32	63	12	ns
	Continuous subcutaneous insulin infusion	36	19	47	9	ns

*p*-value was calculated using Chi Square test.

After 6 years, patients from this cohort were recontacted to undergo clinical questionnaires; 32 answered the call and completed questionnaires. Results of the MNSI and COMPASS-31 questionnaires are shown in Table 4.

**Table 4:** MNSI score and related percentages; percentage of positive responses to the questionnaire, median, first (Q1) and third (Q3) quartiles, mean and standard deviation (SD) of recorded scores.

MNSI Score	1–3	4–5	6–13
Percentage (%)	88.2	11.8	0
Score ≥ 3 (%) (11.8)			
Median	Q1-Q3	Average	SD
1	0–1	0.97	1
COMPASS 31 score	0–10	11–20	≥ 21
Percentage (%)	43.7	40.6	15.7
Median	Q1-Q3	Average	SD
7.52	2.93–18.9	12.01	12.09

A total score ≥ 3 was considered pathological. Compass 31 score and related percentages; median, first (Q1) and third (Q3) quartiles, mean and standard deviation (SD) of the scores recorded at COMPASS 31.

Regarding the Clarke questionnaire, 97% of patients demonstrated normal awareness of hypoglycemic states (A, awareness), while 3% had reduced awareness (R, reduced awareness); none, however, was unaware (U, unawareness). For the modified Clark questionnaire, the score is considered above the threshold if ≥10 and was achieved by 41% of patients (Table 5).

**Table 5:** Clarke score according to categorical scheme (A awareness, R reduced awareness, U unawareness) and related percentages.

Categorical score (%)	A		97		
	R		3		
	U		0		
Modified Clarke score	Median	Q1-Q3	Average	SD	Score ≥ 10
	9	7–10	9.68	4.52	41%

Modified Clarke score and related median, first (Q1) and third (Q3) quartiles, mean and standard deviation (SD). A score ≥ 10 was considered pathological.

The results obtained at the questionnaires were then used to perform a correlation analysis with outcome at NCS, dietary adherence, and the BMI, by linear regression.

The MNSI questionnaire did not produce statistically significant correlations (R2 = 0.022); indeed, the association with the outcome at NCS was not statistically significant (*p*-value = 0.57), and likewise, the adherence to a healthy diet and BMI were not (*p*-value 0.51 and 0.98, respectively). Similarly, the results obtained at COMPASS 31 do not appear to be associated with the variables considered. Specifically, *p*-values of 0.86, 0.95 and 0.63 are observed for the correlation with the outcome at NCS, diet adequacy and BMI, respectively (R2 = 0.008). As Clarke’s questionnaire investigates subjects’ perceptions of hypoglycemic events, a correlation analysis between the number of these events and the variables under investigation was chosen, and then the evaluation of the questionnaire was conducted. The correlation analysis between the number of hypoglycemic events in the past 12 months and the outcome on NCS, dietary adherence, and BMI was conducted by regression with Poisson distribution (R2 = 0.852). Specifically, it was observed that the presence of an abnormal outcome at NCS increases the probability of experiencing severe hypoglycemia by 97.13-fold (*p*-value = 0.009).

In contrast, the correlation between dietary adherence and BMI was not statistically significant (*p*-values of 0.160 and 0.831, respectively). Finally, upon evaluation of the Clarke questionnaire by linear regression (R2 = 0.173), the results obtained did not observe an association with the outcome at NCS, BMI (*p*-value of 0.32 and 0.97, respectively). However, as dietary adherence increased, a reduction of 1.94 points on the questionnaire was observed (*p*-value = 0.04).

The values obtained in the previous correlation analyses are shown in Table 6.

**Table 6:** Correlation analysis between several parameters (NCS outcome, diet adequacy, and BMI) and the score on the questionnaires administered or the increase of severe hypoglycemic episodes.

	β	IC	*p*-value
MNSI			
NCS outcome	0.66	−0.63 − 1.12	0.57
Adequacy of diet	0.15	−0.31 − 0.60	0.51
BMI	−0.00	−0.12 − 0.12	0.98
COMPASS 31			
NCS outcome	0.96	−9.70 − 11.63	0.86
Adequacy of diet	0.16	−5.37 − 5.70	0.95
BMI	−0.34	−1.75 − 1.08	0.63
Hypoglycemic events			
NCS outcome	97.13	3.15–2997.35	0.009
Adequacy of diet	3.73	0.59–23.43	0.160
BMI	1.07	0.57–2.00	0.831
Clarke			
NCS outcome	1.79	−1.85 − 5.44	0.32
Adequacy of diet	−1.94	−3.84 − −0.05	0.04
BMI	0.01	−0.48 − 0.49	0.97

The values of beta coefficient, confidence interval (CI), and *p*-value are reported. The tests used were linear regression and Poisson regression for questionnaires and hypoglycemic events, respectively.

## Discussion

DN is one of the complications of T1DM and is the most frequent form of neuropathy in the general population. It is a prominent condition, especially in adults with diabetes, as it results in a considerable increase in morbidity and mortality, while in the pediatric age, it is rarely seen but may sometimes occur in adolescents with long disease duration (19). Deserving special attention is diabetic autonomic neuropathy (DAN), a condition that can occur within a few years of DM onset and has a broad spectrum of manifestations (6, 20). To identify alterations in somatic and vegetative nerve conduction in the subclinical phase, a uniform screening program for DN is therefore needed.

Puberty is considered a critical period for the development of microangiopathic complications, so adequate glucometabolic values at this stage are imperative to reduce the incidence and slow the course of these manifestations (12).

The 2022 ISPAD Guidelines indicated to begin screening programs for DN at age 11 in subjects with a disease lasting for at least 2–5 years and to repeat it annually. The method consists of a clinical investigation that assesses sensitivity at the foot level, osteo-tendinous reflexes in the lower extremities (especially the Achilles), and the presence of autonomic dysfunction. On the other hand, electrophysiological investigations are not provided and are used only in cases of diagnostic uncertainty due to atypical clinical manifestations (3).

Analysis of motor and sensory nerve conduction by NCS represents the gold standard for the diagnosis of DN as it allows the identification of early signs of neuropathy in subclinical stages of disease and also has high sensitivity and reproducibility (20).

The present study evaluated a cohort of 72 individuals who had previously undergone NCS evaluation and investigated the subsequent clinical evolution over a 6-year period in 32 of them.

The first part of the study compared the NCS outcomes of T1DM patients with those of 18 healthy adolescents in a case–control study. Patients with T1DM showed some differences in terms of MCV of external popliteal and internal popliteal sciatic nerves, SCV of median, ulnar, and sural nerves, and latencies of F-wave and sensory components of median and ulnar nerves. This discrepancy could be considered as the first signal of nerve damage that could be traced only by instrumental investigation.

As already reported by Franceschi et al., the risk factors that mostly determine the evolution of chronic complications are hyperglycemia/HbA1c, age, diabetes duration, the presence of other microvascular complications, diet, waist/height ratio, lipid profile and blood pressure. For this reason, all this information was collected during the follow-up. The same review also demonstrated that the use of questionnaires, and particularly the MNSI, is a valuable tool for the early identification of DN-related symptoms. Indeed, this questionnaire is designed to identify clinical symptoms that can be correlated with the presence of sensorimotor peripheral polyneuropathy (21).

The data collected also included the number of episodes of ketoacidosis (DKA) and severe hypoglycemia that occurred in the past 12 months, as well as the completion of two other questionnaires validated in the literature: the COMPASS31, which allows the identification of the presence of clinically evident autonomic impairment, and the Clarke, which investigates the awareness, frequency, and severity of hypoglycemic episodes. Using these forms allows us to obtain scores that can be correlated with the likelihood of each subject to be affected by DN.

Regarding the MNSI questionnaire, the proportion of patients with an overall high result (≥ 3) was 11.8%; the positivity to this questionnaire should raise suspicion of initial damage to the sensory and motor nerve components. It is worth noting that only a minority of subjects had clinical symptoms. On the other hand, the most frequently reported symptoms were numbness, paresthesia, cramps, and skin dryness in the lower extremities. These manifestations are particularly common in the early stages of DN (22). Therefore, in this subgroup, it might be useful to continue with the follow-up to detect a possible evolution of full-blown forms of DN over a more extended period.

As for COMPASS 31, a relatively small mean score (12.01) and a significant standard deviation (12.09) were observed. This suggests that most patients do not present symptomatology compatible with autonomic impairment, but there is a wide variability.

In more detail, it was observed that the symptom domains with higher scores were orthostatic intolerance (5.53) and gastrointestinal symptoms (3.83). It should be specified that the result obtained from each domain was weighted by the use of a coefficient because the authors of COMPASS 31 considered the association of DAN with some symptoms to be stronger than others (17, 23) orthostatic intolerance in terms of score at COMPASS 31 determines the majority contribution, participating for almost half of the total result, so it is relevant to note that the 35% of the patients show abnormalities affecting this domain. It might be useful to follow these individuals over time and administer the questionnaire again to intercept clear DN manifestations.

Finally, the Clarke questionnaire was evaluated using the modified Clarke score. This choice was made to obtain continuous and not categorical values as in the classic questionnaire score (A, R, U) 0.25 A cut-off value of 10 was chosen as indicated in the work conducted by Kim et al. 18 Using this cut-off, the percentage of subjects with alterations in the recognition of hypoglycemic states was around 41%.

The analysis of the individual responses shows that nearly 80% of the patients experienced hypoglycemic episodes in which they felt confused and disoriented in the past 6 months. However, only about 30% reported no noticeable symptomatology during these events.

To evaluate these results, a correlation analysis was performed to understand whether abnormal outcomes on NCS, adequacy of diet or BMI could explain the scores obtained on the questionnaires. We could not evaluate the correlation with HbA1c due to the lack of data on some participants.

The analysis of the MNSI questionnaire was conducted by linear regression, without significance (R2 = 0.022); again, there was no statistically significant correlation between the variables considered and MNSI outcomes. The lack of correlation with the NCS outcome was probably due to the characteristics of the MNSI questionnaire, which investigates sensorimotor symptoms while participants had predominantly autonomic alterations.

The analysis performed on the COMPASS 31 was also done by linear regression and did not produce statistically significant results (R2 = 0.008). In this case, correlation was probably not observed due to the small sample size, which does not allow for statistically significant results. In addition, it must be considered that SSR is an examination that investigates autonomic pathways destined for skin innervation; COMPASS 31 highlights symptomatology related to these nerve pathways through the evaluation of secretomotor symptoms, which, however, play a marginal role in the overall score of the questionnaire. Finally, it is also possible that the damage to the vegetative system identified by the NCS survey has not progressed sufficiently to result in full-blown DAN within only six years.

Prior to evaluating Clarke’s questionnaire, we chose to analyze the association between the number of hypoglycemic events incurred in the past year and the three variables described in previous correlation studies. Indeed, the frequency of severe hypoglycemia is a relevant parameter in the management of T1DM and correlates directly with the Clarke questionnaire.

In this case, the analysis was conducted using regression with Poisson distribution, and the model used seems to effectively explain the increase in hypoglycemic events (R2 = 0.852). The most significant finding was the strong relationship with outcomes at NCS. The finding of SSR abnormalities increases the risk of experiencing hypoglycemic events by 97-fold (*p*-value = 0.009). In contrast, no relationship was observed between diet adequacy (*p*-value = 0.160) and BMI (*p*-value = 0.831).

One possible explanation for this correlation is related to the reduced perception of hypoglycemia for nerve damage and the consequent increase in the number of severe episodes. In addition, an autonomic impairment may worsen metabolic compensation by increasing glycemic variability and, consequently, the amount of hypoglycemia. It is important to note that the distribution of these episodes among participants was not homogeneous; indeed, a high number of hypoglycemia was recorded in a relatively small number of subjects. It can be inferred that although hypoglycemic events are not frequent in the cohort considered, several subjects were particularly predisposed to the development of severe episodes.

Finally, a correlation analysis was performed between Clarke’s questionnaire score, outcome at NCS, diet adequacy and BMI using linear regression. However, the model used seems only partially to explain the results obtained (R2 = 0.173).

No relationship was observed between questionnaire score and outcome at NCS or BMI (*p*-value 0.32 and 0.97, respectively). In contrast, dietary adequacy seems to play a significant role; indeed, as compliance with the dietary pattern increases, Clarke’s questionnaire score decreases by about 2 points (*p*-value = 0.04).

The absence of a direct relationship with SSR outcome contrasts with the previously described correlation between the same test and the number of hypoglycemic events incurred in the past 12 months. In the literature, however, a close association between the frequency of hypoglycemic episodes and reduced awareness during them was reported (16). It is emphasized that the earliest clinical manifestations of hypoglycemic states involve the adrenergic system, with anxiety, tachycardia, tremors, and algid sweating, which can be impaired in case of damage to the autonomic pathways (24).

We therefore speculate that the low significance of the correlation with the Clarke questionnaire was due to the small number of study participants. It might be interesting to improve the present study by increasing the number of subjects.

In the review by Yu Kuei Lin and co-workers 2020, it is estimated that approximately 25–40% of individuals with T1DM have impaired perception of hypoglycemic states (IAH or Impaired Awareness of Hypoglycemia) (25). The mechanisms leading to the development of IAH have not yet been fully elucidated; dysfunction in the adrenal medulla, alterations in cortisol secretion, or associations with CAN are suspected. The association between the occurrence of hypoglycemia and CAN was evaluated in a study conducted by Dagogo-Jack et al. in which autonomic dysfunction was shown to be responsible for a higher frequency of hypoglycemic events due to both a reduction in predictive ability on the part of the subject and poor metabolic compensation to lower blood glucose levels (26).

The results of the present study are therefore aligned with observations in the literature and identify the possibility of tracing autonomic system damage in the subclinical phase by a nerve conduction study performed in the pediatric age.

This work’s main limitation is the small participant cohort size. The loss of a substantial number of subjects at follow-up reduces the statistical power of the information obtained. It does not allow the different parameters to be evaluated in a multivariate regression that identifies correlations as a whole.

In addition, the comparison has been made on information of different natures; indeed, the results of an instrumental survey have been analyzed, and an association has been sought with clinical information that does not allow an unambiguous and objective comparison. The present work could be improved with the subsequent evaluation of further NCS sensorimotor and SSR examination to identify the evolution of the previously found abnormalities.

## Conclusion

Based on observed data, we can conclude that there is a statistically significant difference between NCS outcomes of healthy adolescents and those affected by T1DM. In addition, the frequency of clinical abnormalities in diabetic subjects was 11.8% for sensory and motor pathways and 41% for autonomic pathways, and no specific alterations were observed on specific tests for diabetes-related neurological complications.

The absence of SSR would seem to predispose subjects with T1DM to more hypoglycemia 6 years later, although such episodes are often experienced in adequate form.

Improving the current management of DN requires numerous interventions in prevention and early diagnosis to intervene early on the factors that determine its onset and promote its progression. Therefore, screening for this complication must begin in the pediatric age, when DN is still in the subclinical stage. Nerve conduction studies, including SSR and major clinical questionnaires, may represent the optimal tool to detect early damage to the somatic and autonomic nervous system.

## Data availability statement

The raw data supporting the conclusions of this article will be made available by the authors, without undue reservation.

## Ethics statement

The studies involving humans were approved by Comitato Etico Interaziendale A.O.U. Citta’ della Salute e della Scienza di Torino – A.O. Ordine Mauriziano di Torino – A.S.L. Città di Torino. The studies were conducted in accordance with the local legislation and institutional requirements. Written informed consent for participation in this study was provided by the participants’ legal guardians/next of kin.

## Author contributions

DT: Conceptualization, Investigation, Project administration, Supervision, Validation, Writing – review & editing. CC: Conceptualization, Data curation, Investigation, Writing – review & editing. CN: Project administration, Supervision, Visualization, Writing – review & editing. DM: Formal analysis, Software, Writing – review & editing. ED: Data curation, Investigation, Writing – original draft. IR: Conceptualization, Methodology, Writing – review & editing. LS: Supervision, Validation, Writing – review & editing.
